# Anti-chlamydial activity of vaginal fluids: new evidence from an *in vitro* model

**DOI:** 10.3389/fcimb.2024.1403782

**Published:** 2024-06-07

**Authors:** Sara Morselli, Camilla Ceccarani, Marielle Ezekielle Djusse, Luca Laghi, Tania Camboni, Clarissa Consolandi, Claudio Foschi, Marco Severgnini, Antonella Marangoni

**Affiliations:** ^1^Section of Microbiology, Department of Medical and Surgical Sciences, Alma Mater Studiorum - University of Bologna, Bologna, Italy; ^2^Institute of Biomedical Technologies, National Research Council, Segrate, Italy; ^3^National Biodiversity Future Center S.c.a.r.l., Palermo, Italy; ^4^Department of Agricultural and Food Sciences, University of Bologna, Cesena, Italy; ^5^Microbiology Unit, IRCCS Azienda Ospedaliero-Universitaria di Bologna, Bologna, Italy

**Keywords:** Chlamydia trachomatis, lactobacilli, vaginal microbiome, metabolome, women’s health

## Abstract

**Introduction:**

We assessed the *in vitro* anti-chlamydial activity of fresh vaginal secretions, deciphering the microbial and metabolic components able to counteract *Chlamydia trachomatis* viability.

**Methods:**

Forty vaginal samples were collected from a group of reproductive-aged women and their anti-chlamydial activity was evaluated by inhibition experiments. Each sample underwent 16S rRNA metabarcoding sequencing to determine the bacterial composition, as well as ^1^H-NMR spectroscopy to detect and quantify the presence of vaginal metabolites.

**Results:**

Samples characterized by a high anti-chlamydial activity were enriched in *Lactobacillus*, especially *Lactobacillus crispatus* and *Lactobacillus iners*, while not-active samples exhibited a significant reduction of lactobacilli, along with higher relative abundances of *Streptococcus* and *Olegusella*. *Lactobacillus gasseri *showed an opposite behavior compared to *L. crispatus*, being more prevalent in not-active vaginal samples. Higher concentrations of several amino acids (i.e., isoleucine, leucine, and aspartate; positively correlated to the abundance of *L. crispatus* and *L. jensenii*) lactate, and 4-aminobutyrate were the most significant metabolic fingerprints of highly active samples. Acetate and formate concentrations, on the other hand, were related to the abundances of a group of anaerobic opportunistic bacteria (including *Prevotella, Dialister, Olegusella, Peptostreptococcus, Peptoniphilus, Finegoldia* and *Anaerococcus*). Finally, glucose, correlated to *Streptococcus, Lachnospira* and *Alloscardovia* genera, emerged as a key molecule of the vaginal environment: indeed, the anti-chlamydial effect of vaginal fluids decreased as glucose concentrations increased.

**Discussion:**

These findings could pave the way for novel strategies in the prevention and treatment of chlamydial urogenital infections, such as lactobacilli probiotic formulations or lactobacilli-derived postbiotics.

## 1 Introduction

*Chlamydia trachomatis* (CT), an obligate intracellular pathogen, represents the most common bacterial sexually transmitted infection (STI) worldwide, with a significant clinic, economic and public health impact (ECDC, 2024; World Health Organization (WHO), 2015). In women, urogenital CT infections (i.e., cervicitis, urethritis) are often characterized by the absence of symptoms, potentially resulting in a range of serious sequelae and complications including pelvic inflammatory disease, ectopic pregnancy, and infertility (Haggerty et al., 2010; Menon et al., 2015).

In recent years, the correlation between CT presence and the microbial/metabolic composition of the vaginal environment has garnered particular attention, elucidating mechanisms involved in the protection from chlamydial infection (Parolin et al., 2018a; Ceccarani et al., 2019; Tamarelle et al., 2019; Raimondi et al., 2021).

Indeed, it has been shown that a vaginal microbiome dominated by certain species of *Lactobacillus* is crucial for preventing CT infections (Nardini et al., 2016; Di Pietro et al., 2022). The protective role of lactobacilli against CT is exerted through different mechanisms that act on both extracellular and intracellular steps of the chlamydial cycle. These include the production of various antibacterial compounds (e.g., lactic acid, hydrogen peroxide, bacteriocins, and biosurfactants), competitive exclusion for epithelial adhesion, and immunomodulation (Younes et al., 2018; Parolin et al., 2018b; Zalambani et al., 2023).

The protective role of lactobacilli is strengthened by the demonstration that a higher risk of CT transmission and acquisition is reported in cases of bacterial vaginosis (BV), a clinical condition characterized by the depletion of *Lactobacillus* and an overgrowth of anaerobic genera such as *Gardnerella, Prevotella, Megasphaera*, and *Atopobium*, has been reported (Wiesenfeld et al., 2003; Abbai et al., 2016). Changes in bacterial communities during BV conditions are accompanied by significant alterations in vaginal metabolite composition, characterized by a decrease in lactate concentration and higher levels of biogenic amines and short-chain organic acids (Ceccarani et al., 2019).

To date, only a few studies have focused on the *in vitro* interaction between vaginal fluids/secretions and CT, and many aspects of the mechanisms involved in the activity against chlamydial infectivity remain to be fully elucidated (Mastromarino et al., 2014; Nardini et al., 2016; Zalambani et al., 2023). Therefore, the aim of the present work was to assess the *in vitro* anti-chlamydial activity of fresh vaginal secretions collected from reproductive-aged women, deciphering the microbial composition (microbiome analysis by 16S rRNA metabarcoding sequencing) and the metabolic components (metabolome analysis by ^1^H-NMR) of the vaginal fluids associated with protection from CT infection. Understanding the microbial/metabolic fingerprints of vaginal fluids related to their activity against CT could pave the way for the development of novel antimicrobial-free strategies for the prevention and treatment of chlamydial urogenital infections.

## 2 Materials and methods

### 2.1 Study cohort and sample collection

Between September and November 2022, 10 women were enclosed in the study. The women were volunteers and were selected from those who had expressed interest in participating in this study. All of them were attending degree courses at the University of Bologna, Italy. At the enrollment, exclusion criteria were: (i) antibiotic use in the month prior to sampling; (ii) menstruating at the time of sampling; (iii) HIV infection; (iv) presence of chronic conditions (e.g., diabetes, autoimmune diseases, malignancies); (v) pregnancy; (vi) age<18 years.

**Figure 1:**
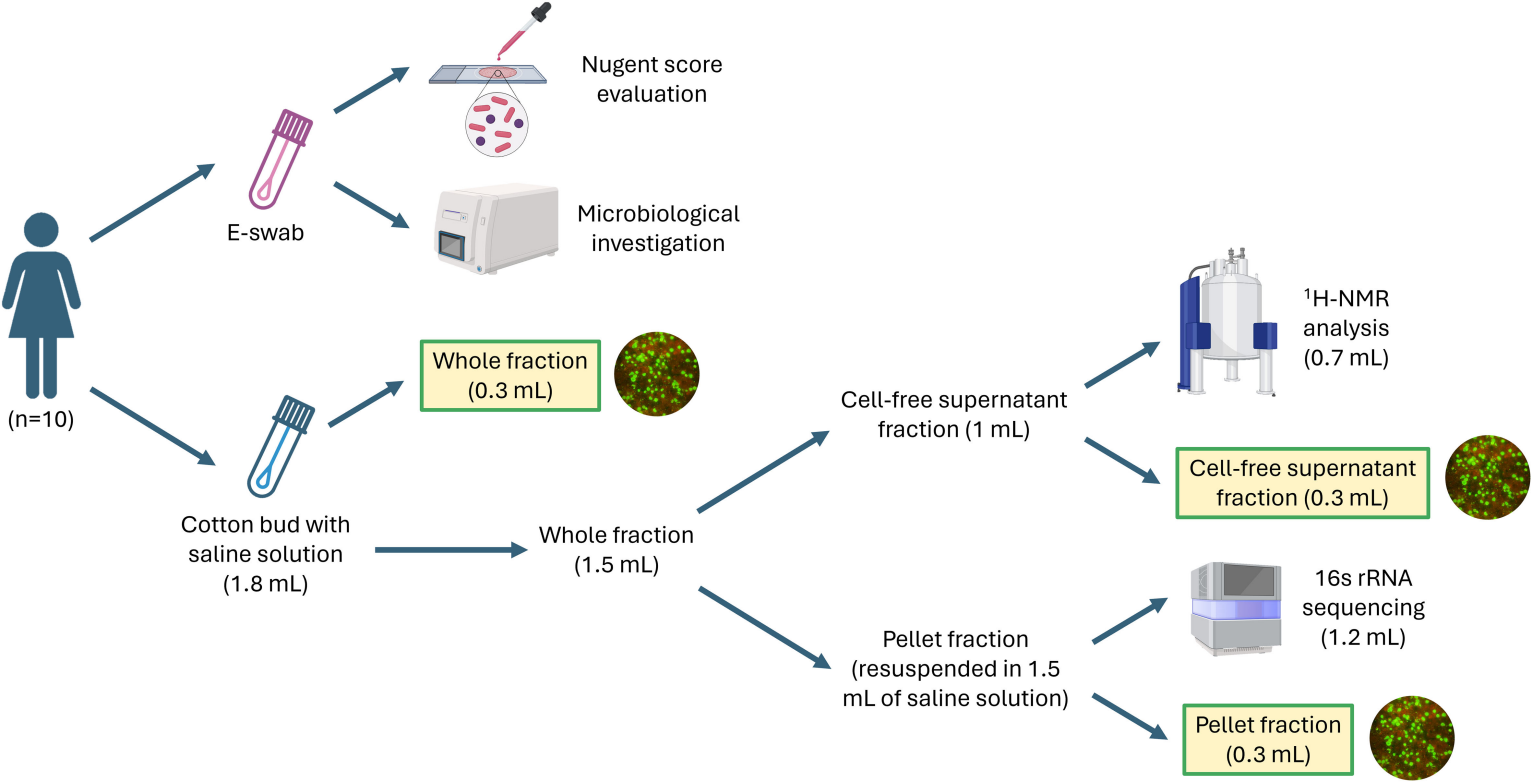
Workflow of the experiments performed in this study. For each woman, the vaginal sampling was performed 4 times during a period of 40 days.

Demographic, clinical, and behavioral data were recorded from each participant. Each woman underwent four self-collected vaginal samplings within 40 days, at four different time points (starting from the first day of menstrual cycle, volunteers were asked to submit their samples at the following time points: 5-7 days, 12-16 days, 21-25 days, 32-40 days). As summarized in **Figure 1**, two vaginal swabs were collected at each time point. At first, the secretions were collected with a sterile cotton bud, and it was immediately re-suspended in 1.8 mL of sterile saline, while, as a second step, secretions were collected with a flocked swab (E-swab Copan, Brescia, Italy). Both swabs were processed within 10 min of collection. The swab suspended in saline was vortexed, and 300 µL of the suspension were sampled to test the anti-chlamydia activity (‘whole fraction’). Subsequently, the remaining suspension was centrifuged at 10,000 × g for 15 min: 300 µL of the supernatant were used to test the anti-chlamydia activity (‘cell-free supernatant fraction’), while 700 µL were preserved at -80°C until the metabolomic analysis by means of ^1^H-NMR spectroscopy (see specific paragraph below). Finally, the residual supernatant portion was discarded, and the pellet was resuspended in 1.5 mL of sterile saline: 300 µL of this pellet solution were used to test anti-chlamydia activity (‘pellet fraction’), while the remaining 1200 µL were used to extract nucleic acids for microbiome analysis (see specific paragraph below).

The E-swab, after being vortexed, was used for both microscopic evaluation and microbiological tests, using, at first, the Nugent score for a preliminary assessment of the vaginal flora composition (Nugent et al., 1991). According to this score, women were grouped as follows: “H” group (normal lactobacilli-dominated microbiota, score 0-3), “I” group (intermediate microbiota/flora, score 4-6), “BV” group (bacterial vaginosis, score 7-10) (Zozaya-Hinchliffe et al., 2010). Moreover, Seeplex STI Master Panels 1 and 3 (Seegene, Seoul, KR) were performed following the manufacturer’s instructions, to investigate the potential presence of the most common urogenital STIs agents (i.e., *Chlamydia trachomatis*, *Neisseria gonorrhoeae*, *Trichomonas vaginalis* and *Mycoplasma genitalium*), and *Candida* spp. A positive result would have led to exclusion from the study.

A written informed consent was obtained from all participants and the Bioethics Committee of the University of Bologna (protocol number 0122421) approved the protocol of the study.

### 2.2 Cell line and *Chlamydia trachomatis* strain

HeLa cells (ATCC^®^ CCL-2) were used as an *in vitro* model for experiments. Cells were grown in individual tubes containing sterile coverslips (Thermo Fisher Scientific, Waltham, MA, USA) in 5% CO_2_ at 37°C. Cells were cultivated in DMEM medium (EuroClone, Pero, Italy), supplemented with 10% fetal bovine serum and 1% L-glutamine, without antibiotics.

*Chlamydia trachomatis* strain GO/86, serovar D, was used for the present study (Marangoni et al., 2015a). This strain was clinically isolated in 1986 from a urethral swab sent to the Microbiology Laboratory of IRCSS Azienda Ospedaliero-Universitaria of Bologna, Italy, for routine diagnostic procedures and it belongs to the laboratory collection. The purification of elementary bodies (EBs) and the evaluation of the infectivity titer (expressed as inclusion forming units IFU/mL) have been described in detail elsewhere (Foschi et al., 2019).

### 2.3 Evaluation of the anti-chlamydial activity of vaginal fluids

To study the ability of the whole vaginal fluids and their fractions (i.e., bacterial pellets and supernatants) to directly counteract CT viability, ‘inhibition experiments’ were performed in triplicate for each woman at each time point, as follows. HeLa cells were seeded in individual tubes and allowed to reach a total cell number of approximately 5 × 10^5^. Afterwards, for each single experiment, 5 × 10^4^ CT EBs were re-suspended for 30 min at 37°C with 5% CO_2_ in four separate solutions containing (i) 100 µL of the whole vaginal fluid, (ii) 100 µL of the vaginal cell-free supernatant, (iii) 100 µL of the vaginal pellet (re-suspended in sterile saline) and (iv) 100 µL of sterile saline (used as controls). At the end of the incubation, the samples were inoculated into HeLa cells, grown in DMEM medium in individual tubes containing sterile coverslips, and centrifuged at 530 × g for 1h. At the end of the centrifugation, culture medium was replaced with fresh DMEM, and HeLa cells were incubated at 37°C with 5% CO_2_ for 48h.

CT infection was estimated by counting the number of IFUs by direct immunofluorescence, using a fluorescein-conjugated anti-chlamydial LPS monoclonal antibody (Meridian, Cincinnati, OH, USA). Slides were observed under an epi-fluorescence microscope (Eclipse E600, Nikon, Tokyo, Japan). The number of IFUs was counted in 30 randomly chosen 200× microscopic fields.

Results were expressed as the percentage (average percentage ± standard deviation) of CT infectivity, comparing the number of IFUs from the individual experiments to the control tubes (number of IFU/field of the controls ranged between 60 and 85). Tested fractions were arbitrarily divided into three groups based on their anti-chlamydial activity: ‘High activity’ (infectivity reduction of 61-100% compared to control), ‘Intermediate activity’ (infectivity reduction of 41-60% compared to control), and ‘No activity’ (infectivity reduction of 0-40% compared to control).

### 2.4 Microbiome analysis

DNA extraction from vaginal swabs was carried out by means of the Versant molecular system (Siemens Healthcare Diagnostics, Tarrytown, NY, USA) (Marangoni et al., 2015b). Afterwards, the V3-V4 hypervariable regions of the bacterial 16S rRNA gene were amplified, according to the 16S metagenomic sequencing library preparation protocol (Illumina, San Diego, CA). Final indexed libraries were prepared in a equimolar (4 nmol/L) pool, then denaturation and dilution to 6 pmol/L were performed, before loading onto the MiSeq flow cell (Illumina) (Severgnini et al., 2021). Sequencing was performed by a paired 2 × 300 bp run.

Amplicon sequence variants (ASVs) were identified from 16S paired-end sequencing using the Divisive Amplicon Denoising Algorithm (DADA2, version 1.18.0; Callahan et al., 2016) pipeline, including filtering and trimming of the reads. Reads per sample were trimmed to 6,800 reads in order to compensate for the sequencing unevenness of the samples and to provide a consistent minimum amount for the downstream analysis, carried out through the “phyloseq” package (version 1.34.0) (McMurdie and Holmes, 2013). Alpha-diversity evaluation was performed according to several microbial diversity metrics (i.e., Chao1, Shannon Index, Observed Species); the Faith’s phylogenetic tree diversity metric (“PD whole tree”) was elaborated through the “btools” package (https://github.com/twbattaglia/btools). Beta-diversity analysis was conducted using the unweighted Unifrac distance and the principal coordinates analysis (PCoA).

Taxonomy was assigned to the ASVs using the 8-mer-based classifier from the 11.5 release of the RDP database and using the GTDB 16S rRNA database (release r207) (Parks et al., 2022).

*Lactobacillus* species-level characterization was performed as in Severgnini et al. (2022), by BLAST-aligning all reads belonging to the Lactobacillaceae family to a custom reference database made up collecting all available reference sequences in NIH-NCBI database (ftp://ftp.ncbi.nlm.nih.gov/genomes/GENOME_REPORTS/prokaryotes.txt) of 17 species commonly found in the vaginal environment. The dataset included 2,403 sequences belonging to 17 species and 6 genera (i.e., *Lacticaseibacillus*, *Lactiplantibacillus*, *Lactobacillus*, *Levilactobacillus*, *Ligilactobacillus*, and *Limosilactobacillus*). Potential matches were filtered in order to retrieve an unequivocal classification for each read.

### 2.5 Metabolome analysis

Metabolomic analysis was performed by means of a ^1^H-NMR spectroscopy: 100 μL of a D_2_O solution of 3-(trimethylsilyl)-propionic-2,2,3,3-d4 acid sodium salt (TSP) 10 mM set to pH 7.0 were added to 700 µL of the cell-free supernatants of the vaginal swabs. ^1^H-NMR spectra were recorded at 298 K with an AVANCE III spectrometer (Bruker, Milan, Italy), operating at a frequency of 600.13 MHz, equipped with Topspin software (Ver. 3.5) (Foschi et al., 2018). The signals originating from large molecules were suppressed by a CPMG filter of 400 spin-echo periods, generated by 180° pulses of 24 μs separated by 400 μs (Ventrella et al., 2016). To each spectrum, line broadening (0.3 Hz) and phase adjustment were applied by Topspin software, while any further spectra processing, molecules quantification and data mining step were performed in R computational language (version 4.0.5) by means of in house-developed scripts. The spectra were aligned towards the TSP signal, set at −0.017 ppm in agreement with Chenomx software data bank (version 8.3, Chenomx Inc., Edmonton, Alberta, Canada). The spectra were then baseline-adjusted by means of peak detection according to the “rolling ball” principle implemented in the “baseline” R package (Kneen and Annegarn, 1996; Liland et al., 2010). The signals were assigned by comparing their chemical shift and multiplicity with Chenomx software data bank. Molecules were quantified in the first sample acquired by employing the added TSP as an internal standard. To compensate for differences in sample amount, any other sample was then normalized to such sample by means of probabilistic quotient normalization (Dieterle et al., 2006). Integration of the signals was performed for each molecule by means of rectangular integration.

### 2.6 Statistical analysis

The Fisher-Freeman-Halton test, an extension to Fisher’s exact test, was employed to determine the association between the classification of the samples based on the anti-chlamydial activity and that on the Nugent score (Freeman and Halton, 1951).

For microbiome analysis, statistical evaluation of the alpha-diversity indices was performed by a two-sample Mann-Whitney U-test, whereas beta-diversity differences were assessed by a permutation test with pseudo F-ratios (“adonis” test) (http://CRAN.Rproject.org/package=vegan). Comparisons of microbial relative abundance and metabolite quantities were performed using the Dunn’s test for multiple comparisons (Kruskal-Wallis test), with the Bonferroni or Benjamini-Hochberg corrections for multiple testing. An adjusted p-value <0.05 was considered as statistically significant.

Metabolite concentrations were correlated to bacterial composition by calculating Spearman’s correlation coefficient between metabolites and bacterial genera present ≥1% in at least 1 sample. We performed a Spearman’s rank-based correlation between genus relative abundances and metabolite quantities, selecting only those with p-value<0.05 (i.e., correlation significantly different from 0). To better visualize patterns of positively correlated bacteria and metabolites, a heatmap was drawn, clustering correlation coefficients for metabolites and bacteria (using Pearson’s correlation as clustering metric and average linkage).

### 2.7 Data availability

Raw sequencing data of 16S rRNA gene are available at NCBI Short-reads Archive (SRA) with BioProject accession number PRJNA1062308 (https://www.ncbi.nlm.nih.gov/sra/PRJNA1062308). Raw metabolomic data are available as a **Supplementary Material (Data Sheet S1)**.

## 3 Results

### 3.1 Study population and anti-chlamydial activity of vaginal secretions

Ten reproductive-aged (mean age of 27.5 ± 2.6 years) normal-weight (mean BMI of 20.0 ± 1.3) women were included in the study. All the participants denied the use of hormonal contraceptives, and only one reported a smoking habit of approximately five cigarettes/day. Detailed information for each participant is reported in **Supplementary Table 1**.

The 40 vaginal samples (i.e., 4 samples per subject), collected throughout the study period, were categorized based on microscopic Nugent score, as follows: 30 (75%) showed a lactobacilli-dominated composition (‘H’ group), 3 (7.5%) were characterized by an intermediate flora (‘I’ group), while the remaining 7 (17.5%) harbored a BV-associated microbial composition (‘BV’ group). Two participants in the latter group reported burning sensations and white-grey discharges throughout the entire study period.

The anti-chlamydial activity of each vaginal specimen was tested in an *in vitro* model. At first, to mimic what happens in the natural course of chlamydial infection, we assessed the ability of the whole vaginal sample to counteract CT viability. Subsequently, in order to understand the components associated to the activity against CT, for each sample, we also tested (i) the cellular fraction (i.e., bacterial pellet), and (ii) the supernatant (i.e., cell-free component).

By means of preliminary experiments, we excluded a toxic/cytolytic effect of vaginal pellets on Hela cells (data not shown).

Samples were categorized into three groups based on the extent of the reduction in CT viability. Overall, 11/40 vaginal supernatants were highly active against CT, 15/40 showed intermediate activity, whereas 14/40 were not active. Regarding pellet fractions, 19/40 were classified as highly active, 15/40 as intermediate active, and 6 as not active. The detailed results are reported in **Supplementary Table 2**. Classification of the pellets according to their anti-chlamydial activity was not independent of the vaginal status of the women, as determined by the Nugent score. In fact, all 19 pellets with high anti-CT activity and the majority (10 out of 15) with intermediate activity belonged to the ‘H’ group (lactobacilli-dominated women), whereas not-active pellets were, in 5 out of 6 cases, from BV women. These differences were highly significant (p<0.001, Fisher-Freeman-Halton test); as a matter of fact, the two variables could not be considered independent and disentangled.

### 3.2 Microbiome composition: association with the anti-CT activity

The cellular fraction (i.e., pellet) of each vaginal sample was subjected to 16S rRNA metabarcoding sequencing to determine the bacterial composition. High-throughput sequencing generated 1,861,458 read pairs (average: 46,536 ± 26,151) over the 40 samples. After filtering, denoising, and chimera removal, 858,455 reads (average: 21,461 ± 9,329; range: 7,967 - 44,830) were obtained, forming a total of 1,130 amplicon sequence variants (ASVs).

**Figure 2:**
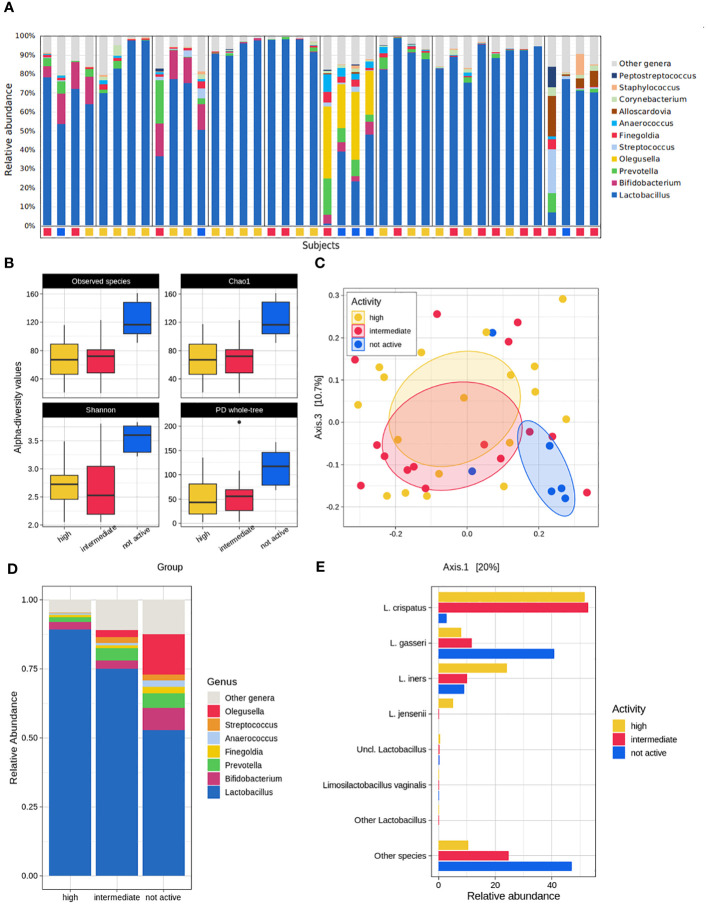
Microbiome analysis. **(A)** Overall composition of the dataset. Stacked bars show the genus-level relative abundance for each patient, subdivided by the different sampling time-points, labelled by their pellet activity classification (yellow = high; red = intermediate; blue = not active). **(B)** Alpha-diversity analysis of the fluid activity evaluation according to the Observed species, Chao1, Shannon index, PD whole tree metrics. **(C)** Principal coordinates analysis reporting the dataset’s beta-diversity for each fluid activity group. **(D)** Genus-level mean relative abundance for high, intermediate, and not active groups. **(E)** Species-level characterization of the *Lactobacillus* genus abundance according to the fluid anti-CT activity subgroups.

The average bacterial composition across all the samples at the phylum level was dominated by Firmicutes (85.7% ± 18.1%), Actinobacteria (9.3% ± 13.0%), Bacteroidetes (3.4% ± 5.2%), and Proteobacteria (1.0% ± 1.7%), which accounted for >99% of the overall average relative abundance. At the genus level, as expected, *Lactobacillus*, which accounted for up to 78.5% on average, comprised most of the composition, with minor contributions from *Bifidobacterium*, *Prevotella*, and *Olegusella* (average rel. ab. in the range 3.0% - 3.5%), *Streptococcus*, *Finegoldia* and *Anaerococcus* (average rel. ab. in the range of 1.0% - 1.2%) (**Figure 2A**).

The bacterial community from highly and intermediately active pellets was consistently characterized by fewer species than that from not-active pellets (p ≤ 0.01 for both high and intermediate vs. not-active for Chao1, Observed species, Shannon, and PD whole tree metrics). Similarly, the bacterial profiles resulted clearly separated in the PCoA plots (p=0.043, intermediate vs. not active, unweighted UniFrac distance; p=0.012, high vs. not active, unweighted UniFrac distance; p=0.005, high vs. not active, weighted UniFrac distance) (**Figures 2B, C**).

The composition of highly and intermediately active pellets was quite comparable, with a non-significant tendency in the latter group towards a reduction in members of the genus *Lactobacillus* (mean rel. ab.: 89.3% vs. 75.1%, in high and intermediate, respectively) and an increase in the genera *Olegusella* (<0.1% vs. 2.6%, respectively) and *Streptococcus* (0.3% vs. 2.1%). On the other hand, deep changes in the microbial signatures were evident in not-active pellets, which, compared to the highly active ones, showed a significant reduction in *Lactobacillus* (mean rel. ab: 89.3% vs. 52.9%, in the high and not-active groups, respectively, p=0.006), a significant increase in *Streptococcus* (mean rel.ab: 0.3% vs. 2.1%, in the high and not-active groups, respectively, p=0.002) and *Olegusella* (mean rel. ab: <0.1% vs. 14.5% in the high and not-active groups, respectively; p=0.010). The same differences were highlighted in the comparison between the microbial profiles of intermediately- and not-active pellets, together with an increase in *Bifidobacterium* (p=0.030), *Finegoldia* (p=0.049), and *Anaerococcus* (p=0.020) in not-active pellets. Among *Bifidobacterium*, a robust share (corresponding to 1.3%-2.4% of the average abundance) was due to *B. vaginalis*, a recently proposed reclassification of *Gardnerella vaginalis* (unpublished data, presented by Barisic et al, 2019; Using Whole-Genome Sequencing to Revise the Classification of Bifidobacterium and Gardnerella Genera; available at: https://delivery-files.atcc.org/api/public/content/255989-Using-WholeGenome-Sequencing-to-Revise-the-Classification-of-the-Bifidobacterium-and-Gardnerella-Gen) as contained in the 16S rRNA database (i.e., GTDB) used for the taxonomic classification of our samples (**Figure 2D**).

Interestingly, a notable difference in the bacterial species belonging to the *Lactobacillus* genus could be observed, since *L. crispatus* was significantly more abundant in highly- and intermediately active pellets (mean rel. ab. >50.0%) than in not-active ones (mean rel. ab. 2.8%), whereas the opposite was observed for *L. gasseri* (mean rel. ab. <12.0% vs 40.8% in high/intermediate and not-active pellets, respectively). *L. jensenii* (mean rel. ab. 5.0 vs <=0.1% in high and intermediate/not-active pellets, respectively) represented a hallmark of highly active pellets (**Figure 2E**; **Supplementary Table 3**). As expected, the microbiome profiles of samples stratified on the basis of the Nugent score largely resembled those based on the anti-chlamydial activity. Samples from women with BV were characterized by higher biodiversity (p<0.02 for all the tested alpha-diversity metrics) and a different microbial profile (p=0.018 and p=0.006 for the unweighted and weighted Unifrac distances, respectively) than those from healthy women (H). A difference was somehow evident also for the women classified as having an “intermediate” vaginal community, which resulted statistically separated from both H and BV women for both the unweighted and the weighted UniFrac distance-based profiles (p=0.033 and p=0.012 for I vs H and I vs BV, respectively). As reported above, the *Lactobacillus* abundance was a hallmark of the H group, as well as *Olegusella* and *Streptococcus* abundances were fingerprints of BV condition (**Supplementary Figure 1**).

### 3.3 Association between anti-chlamydial activity and vaginal metabolites

Fifty-seven metabolites were detected and quantified by ^1^H-NMR spectroscopy on the vaginal fluid supernatants. These molecules mainly belong to groups of short-chain fatty acids (SCFAs), organic acids, amino acids, and biogenic amines (**Supplementary Data Sheet S1**). **Table 1** lists the metabolites whose concentrations differed significantly between samples, stratified by anti-chlamydial activity.

**Table 1:** Concentration (mM) of vaginal metabolites determined by ^1^H-NMR spectroscopy, stratified by the anti-chlamydial activity.

Metabolites	High activity (n=11)	Intermediate activity (n=15)	No activity (n=14)	*p value*	High activity vs Intermediate	High activity vs No activity	Intermediate vs No activity
**4-Aminobutyrate**	0.17 ± 0.19	0.091 ± 0.13	0.0074 ± 0.01	0.0092		**	
**Aspartate**	0.098 ± 0.026	0.072 ± 0.023	0.055 ± 0.018	0.0001		****	
				0.0147	*		
**Glucose**	0.038 ± 0.042	0.071 ± 0.062	0.37 ± 0.40	0.0044		**	
				0.0055			**
**Glutamine**	0.12 ± 0.055	0.15 ± 0.044	0.20 ± 0.090	0.0221		*	
**Isoleucine**	0.13 ± 0.030	0.11 ± 0.037	0.079 ± 0.037	0.0045		**	
**Lactate**	8.46 ± 1.79	7.45 ± 2.22	4.57 ± 1.47	0.0001		****	
				0.0005			***
**Leucine**	0.32 ± 0.060	0.26 ± 0.086	0.19 ± 0.090	0.0009		***	
**Sarcosine**	0.030 ± 0.014	0.022 ± 0.015	0.008 ± 0.0045	0.0002		***	
				0.014			*
**Taurine**	0.39 ± 0.10	0.44 ± 0.088	0.55 ± 0.16	0.008		**	
**Tyrosine**	0.107 ± 0.021	0.090 ± 0.027	0.071 ± 0,030	0.0073		**	
**Valine**	0.15 ± 0.032	0.13 ± 0.032	0.11 ± 0.038	0.038		*	

Results are reported indicating mean ± standard deviation. Asterisks indicate significant variations (p<0.05, after Benjamini-Hochberg correction) in metabolites concentration between groups. Differences were evaluated by Kruskal-Wallis test followed by Dunn’s Multiple Comparison test. *p<0.05 **p<0.01 ***p<0.001 ****p<0.0001.

Except for aspartate, there were no discernible changes in the metabolic profiles of the highly active and intermediately active samples overall. We noticed that a high anti-CT activity was associated with significantly higher levels of 4-aminobutyrate, aspartate, isoleucine, lactate, leucine, sarcosine, tyrosine, and valine, compared to not-active samples. The concentration of glucose tended to increase from the highly active (lowest levels) to the not-active samples (highest levels).

### 3.4 Correlation between vaginal microbiome and metabolome

**Figure 3:**
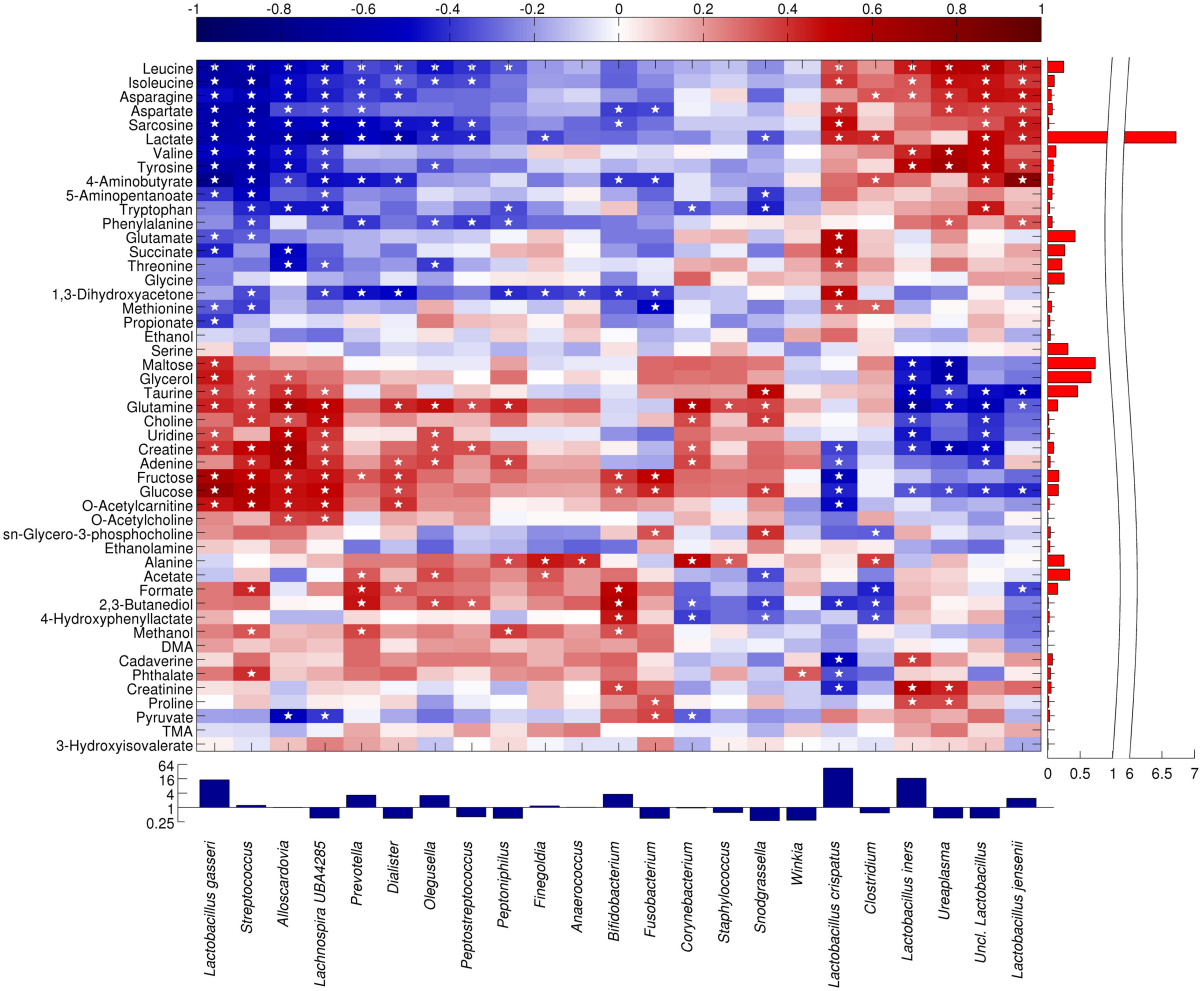
Heatmap showing the Spearman correlation between the main microbial taxa and metabolites. Only taxa present at >1% in at least 10% of the samples are displayed. Statistically significant correlations (p<0.05) are indicated by a star. Both taxa and metabolites are clustered according to Pearson’s correlation and average linkage. For representation purposes, taxa abundance is shown in a log_2_ scale, whereas lactate abundance scale is split.

We performed a correlation analysis aimed at relating the vaginal microbial composition to the metabolite concentrations using Spearman’s rank correlation to determine monotonically increasing or decreasing relationships (**Figure 3**).

A group of bacteria, primarily lactobacilli plus *Ureaplasma* and *Clostridium*, showed a positive correlation with several amino acids (leucine, isoleucine, asparagine, valine, tyrosine, tryptophan, phenylalanine, and aspartate), their degradation products (sarcosine and 5-aminopentanoate), lactate, and 4-aminobutyrate. In particular, the highly abundant metabolite lactate (lactic acid) was significantly correlated to *L. crispatus* (r=0.432), *L. jensenii* (r=0.431), and other unclassified members of *Lactobacillus* genus (r=0.502); moreover, leucine (r between 0.345 and 0.568) and isoleucine (r comprised between 0.333 and 0.470) were positively correlated to *L. crispatus*, *L. iners*, *L. jensenii*, unclassified members of *Lactobacillus*, and *Ureaplasma*.

On the other hand, a further *Lactobacillus* species, *L. gasseri* showed a correlation pattern more similar to that of bacteria from *Streptococcus*, *Lachnospira* and *Alloscardovia* genera, with a high positive correlation to sugars (maltose, r=0.427; fructose, r= 0.608; glucose, r=0.709), glycerol (r=0.459), taurine (r=0.389), glutamine (r=0.435), uridine (r=0.351), creatinine (r=0.399), and o-acetylcarnitine (ρ=0.465). In general, all the interactions between *L. gasseri* and the metabolites were inversely correlated with those of *L. crispatus* and *L. jensenii*.

Finally, a third group of bacterial taxa (i.e., *Prevotella*, *Dialister*, *Olegusella*, *Peptostreptococcus*, *Peptoniphilus*, *Finegoldia* and *Anaerococcus*) showed a moderate positive correlation to alanine (r between 0.236 and 0.455), acetate (r between 0.138 and 0.353), formate (r between 0.345 and 0.568), and 2,3-butanediol (r between 0.120 and 0.466). A non-significant trend towards a positive correlation was also observed for dimethylamine (DMA), cadaverine, and phthalate.

## 4 Discussion

In healthy reproductive-aged women, the vaginal microbiome, generally, shows a predominance of members of the *Lactobacillus* genus and most women display the prevalence of one species among *L. crispatus, L. iners, L. jensenii*, and *L. gasseri* (Ravel et al., 2011). Vaginal resident lactobacilli act as a line of defense against both exogenous and endogenous pathogens, through different mechanisms, including the use of nutrients, establishment and maintenance of low pH levels, and production of antimicrobial compounds, as bacteriocins, peroxides, and organic acids (Valore et al., 2002; Parolin et al., 2018a).

In the case of BV, the abundance of vaginal *Lactobacillus* may decrease significantly, causing an increase in pH levels and the proliferation of different anaerobes, such as *Gardnerella* and *Prevotella* (Coudray and Madhivanan, 2020). This dysbiotic condition is usually accompanied by a peculiar metabolic fingerprint associated with a higher risk of STI acquisition, including CT infection (Coudray and Madhivanan, 2020).

Thus, the evaluation of the microbial and metabolic components of the vaginal fluids capable of either protecting or favoring CT infection is particularly intriguing.

To the best of our knowledge, this is the first study assessing the anti-chlamydial activity of fresh vaginal fluids, investigating the vaginal bacterial taxa and molecules associated with a significant reduction/abolishment of CT viability. Firstly, we found that a vaginal microbiome enriched in *Lactobacillus*, especially *L. crispatus* and *L. iners*, was associated with a significant anti-chlamydial activity. This microbial fingerprint was accompanied by high levels of lactate, leucine, and isoleucine; these metabolites are typically found in lactobacilli-dominated vaginal environments (Foschi et al., 2022).

It is well known that *L. crispatus* represents the hallmark of vaginal health and eubiosis, being indicated as one of the most active species against several urogenital pathogens, including CT (Nardini et al., 2016; Parolin et al., 2018a; Chen et al., 2022). This *Lactobacillus* species is able to produce significant levels of lactate, which is believed to play a central role in host defense against CT, acting on both the extracellular and intracellular phase of chlamydia developmental cycle (Edwards et al., 2019; Zalambani et al., 2023).

On the other hand, *L. iners* has been usually considered as a ‘transitional’ species, characterized by poor activity against urogenital pathogens (Petrova et al., 2015). Although a *L. iners*-dominated vaginal microbiota may be suboptimal compared with a *L. crispatus*-one for CT infection, it has been recently shown that *L. iners* is the most common species in a large subset of women worldwide, being its presence associated with unprotected sex practices and younger age (France et al., 2020; Novak et al., 2022; Carter et al., 2023). Thus, the relatively young age of our cohort could explain the findings of the present study. Nevertheless, further investigations are needed to better understand the impact of *L. iners* on common cervicovaginal infections.

Interestingly, we observed that *L. gasseri* displayed an opposite behavior compared to *L. crispatus*, being more present in not-active samples and correlated to glucose, taurine, and glutamine, three metabolites more abundant in vaginal secretions with no activity against CT.

Several works underline that, even in presence of a strain-specific activity, *L. gasseri* exerts a reduced and more heterogenous spectrum of activity against CT, compared to *L. crispatus* (Nardini et al., 2016; Argentini et al., 2022).

Other interesting data emerged when looking at microbial/metabolic features of vaginal fluids associated with a poor protection from CT infection.

Not-active vaginal samples presented a significant reduction in *Lactobacillus*, along with a significant increase in *Streptococcus* and *Olegusella*. This latter Gram-positive bacterium was recently described as a new member of the Coriobacteriaceae family and was isolated from the vaginal flora of a patient with vaginal dysbiosis (Diop et al., 2016). *Olegusella*, along with other anaerobes such as *Gardnerella*, *Finegoldia*, and *Anaerococcus*, takes part to the polymicrobial environment typically found during BV, characterized by high levels of SCFAs (e.g., acetate, formate) and biogenic amines (i.e., DMA, cadaverine) (Ceccarani et al., 2019; Laghi et al., 2021).

In our experimental model, glucose represented a key metabolite, with vaginal samples with the highest levels of glucose showing the poorest anti-CT activity, whereas highly active samples were characterized by the lowest levels of glucose. This finding could be ascribed to several aspects: (i) the increased glucose consumption by some vaginal *Lactobacillus* strains is associated with a significant reduction in chlamydial elementary bodies infectivity (Nardini et al., 2016), (ii) sugars, as glucose, can increase CT infectivity and virulence, by modulating the expression/exposure of chlamydial membrane ligands (Marziali et al., 2020).

We are aware of some limitations in the present study. Firstly, information about the hormonal levels of participants and their sexual practices was not available. Therefore, larger studies with more detailed data are necessary to elucidate the impact of clinical and behavioral factors on the vaginal inhabitants.

Moreover, examining additional components of the vaginal environment, such as the presence of antibacterial peptides, enzymes, pro/anti-inflammatory cytokines, and microbial biosurfactants, will be crucial to better understand the factors associated with the anti-chlamydial effect.

In conclusion, we have highlighted the microbial and metabolic components of fresh vaginal fluids capable of counteracting CT viability in an *in vitro* model. These findings could pave the way for novel strategies to prevent or counteract chlamydial infection. Among them, we can hypothesize the development of new probiotic formulations based on *L. crispatus* species, the use lactobacilli-derived postbiotics, or the application of vaginal gels containing lactic acid (Pendharkar et al., 2023; Shen et al., 2023; Spaggiari et al., 2023). Finally, we can speculate that the practice of vaginal microbiome transplantation (VMT) from a donor with a flora dominated by *L. crispatus* could be of aid in preventing CT infections, by the restoration of a vaginal healthy state (Ma et al., 2019).

## Data availability statement

The datasets presented in this study can be found in online repositories and/or in the **Supplementary Material**. The names of the repository/repositories and accession number(s) can be found in the article.

## Ethics statement

The studies involving humans were approved by Bioethics Committee of the University of Bologna (protocol number 0122421). The studies were conducted in accordance with the local legislation and institutional requirements. The participants provided their written informed consent to participate in this study.

## Author contributions

SM: Writing – original draft, Investigation, Data curation, Formal analysis. CaC: Writing – original draft, Investigation, Data curation, Formal analysis, Software. MD: Writing – original draft, Formal analysis. LL: Writing – original draft, Formal analysis, Resources. TC: Writing – original draft, Formal analysis, Resources. ClC: Writing – original draft, Formal analysis, Resources. CF: Conceptualization, Writing – original draft, Supervision, Validation. MS: Investigation, Writing – original draft, Data curation, Supervision. AM: Conceptualization, Writing – original draft, Supervision, Validation, Resources.
