# Supplementary material for: Anti-chlamydial activity of vaginal fluids: new evidence from an in vitro model
Source: Front Cell Infect Microbiol. 2024 Jun 7;14:1403782. doi: 10.3389/fcimb.2024.1403782 (PMC11193362; doi:10.3389/fcimb.2024.1403782)
Supplement: Supplementary file 2 [file DataSheet_2.docx]

**Supplementary Table 1. Demographic/behavioral information of the participants.**

| **Woman** | **Age** | **Ethnicity** | **BMI** | **Smoking** | **Contraceptives** |
| --- | --- | --- | --- | --- | --- |
| **1** | 24 | white | 21.1 | no | no |
| **2** | 24 | black | 21.1 | no | no |
| **3** | 28 | white | 18.8 | no | no |
| **4** | 30 | white | 20.6 | no | no |
| **5** | 27 | white | 19.9 | no | no |
| **6** | 27 | white | 18.6 | no | no |
| **7** | 28 | white | 20.6 | no | no |
| **8** | 28 | white | 22.0 | about 5 cigarettes/day | no |
| **9** | 33 | white | 18.6 | no | no |
| **10** | 26 | white | 18.6 | no | no |

**Supplementary Table 2**. **Vaginal samples classified by the anti-CT activity**. For each vaginal sample, the activity against CT is reported for (i) the whole sample, (ii) the cellular fraction (pellet) and (iii) the cell-free component (supernatant). Moreover, the information about the vaginal status classified by the Nugent score is reported (i.e., H=lactobacilli-dominated microbiome; I=intermediate flora; BV=bacterial vaginosis).

|  | **Anti-CT activity** | | |  |
| --- | --- | --- | --- | --- |
| **sample** | **Whole sample** | **Cellular fraction (pellet)** | **Supernatant** | **Vaginal status** |
| A1 | intermediate | intermediate | not active | H |
| A2 | not active | not active | not active | H |
| A3 | intermediate | intermediate | not active | H |
| A4 | intermediate | high | not active | H |
| B1 | high | high | high | H |
| B2 | high | high | intermediate | H |
| B3 | high | high | high | H |
| B4 | high | high | high | H |
| C1 | intermediate | intermediate | not active | H |
| C2 | high | high | intermediate | H |
| C3 | high | high | intermediate | H |
| C4 | not active | not active | not active | BV |
| E1 | high | high | high | H |
| E2 | high | high | high | H |
| E3 | high | high | high | H |
| E4 | high | high | intermediate | H |
| G1 | high | intermediate | high | H |
| G2 | intermediate | intermediate | intermediate | H |
| G3 | high | high | high | H |
| G4 | high | high | intermediate | H |
| H1 | intermediate | intermediate | intermediate | I |
| H2 | not active | not active | not active | BV |
| H3 | not active | not active | not active | BV |
| H4 | not active | not active | not active | BV |
| I1 | high | high | high | H |
| I2 | high | intermediate | intermediate | H |
| I3 | intermediate | high | intermediate | H |
| I4 | high | high | intermediate | H |
| L1 | high | high | high | H |
| L2 | high | intermediate | intermediate | H |
| L3 | high | high | intermediate | H |
| L4 | high | intermediate | intermediate | H |
| M1 | high | intermediate | intermediate | H |
| M2 | high | high | high | H |
| M3 | high | intermediate | intermediate | H |
| M4 | intermediate | intermediate | not active | I |
| N1 | intermediate | intermediate | not active | I |
| N2 | intermediate | not active | not active | BV |
| N3 | intermediate | intermediate | not active | BV |
| N4 | intermediate | intermediate | not active | BV |

**Supplementary Figure 1. Microbiome analysis.** Biodiversity and taxonomic characterization of women with lactobacilli-dominated microbiome (H), intermediate flora (I) or bacterial vaginosis (BV). (A) Alpha-diversity metrics, that is, Chao1, Observed Species, Shannon index, and Faith's PD (PD whole tree); (B) Unweighted Unifrac distance PCoA analysis. Solid dots represent single samples, empty circles are the median centroid per each group, and ellipses report the groups' distribution confidence; (C) Bacterial genera relative abundances. Only genera with greater than 1% of relative abundance in each group are reported.

**
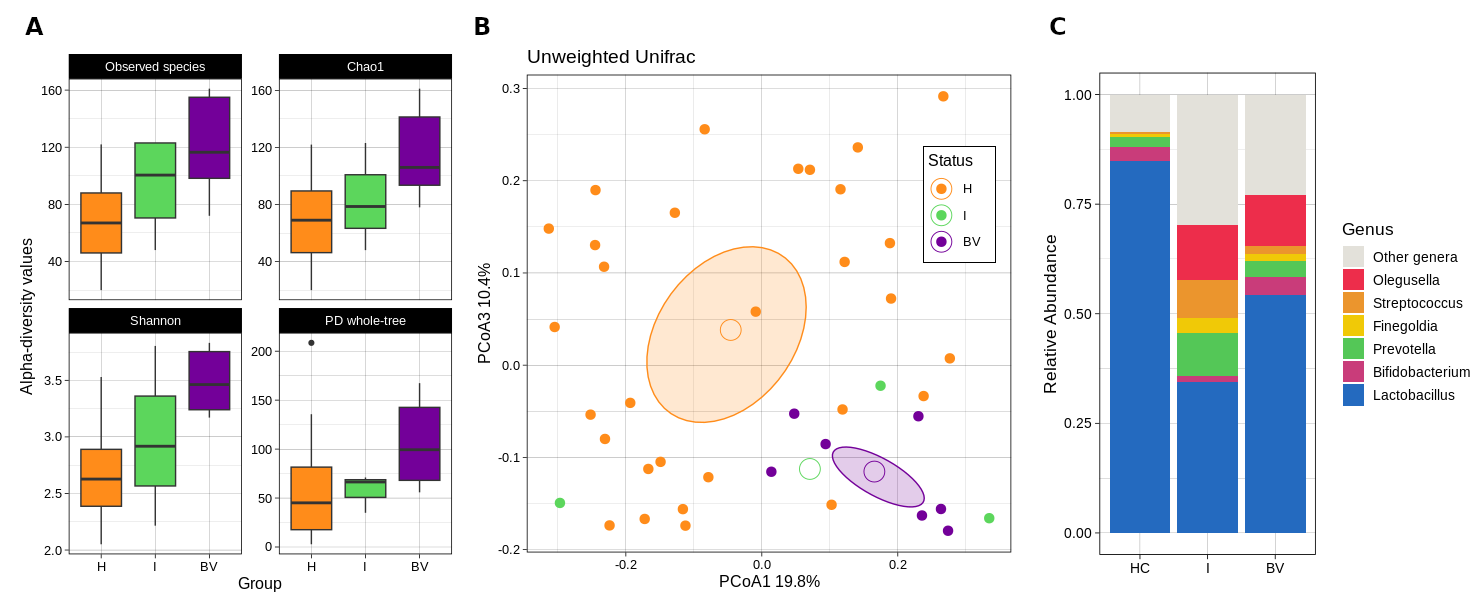
**

**Supplementary Table 3. Species-level characterization of the *Lactobacillus* genus**. The table reports the average (standard deviation) abundance of the main species within the genus *Lactobacillus*. “Uncl. *Lactobacillus*” represents the fraction of bacteria that were characterized only at genus level. “Other *Lactobacillus*” sums up the abundances of the *Lactobacillus* species not included in the table. “Other” is the relative abundance of all the other bacterial genera.

| **Taxa** | **Average rel. ab. (st.dev)** | | | **p-values^a^** | | | |
| --- | --- | --- | --- | --- | --- | --- | --- |
|  | **High** | **Intermediate** | **Not active** | **Overall** | **High**  **Vs**  **Intermediate** | **High**  **Vs**  **Not Active** | **Intermediate**  **Vs**  **Not Active** |
| *Lactobacillus crispatus* | 51.7 (41.1) | 52.9 (48.5) | 2.8 (6.8) | 0.055 | 0.500 | 0.024* | 0.009** |
| *Lactobacillus gasseri* | 8.0 (23.6) | 11.8 (25.5) | 40.8 (27.4) | 0.001*** | 0.054 | <0.001*** | 0.010* |
| *Lactobacillus iners* | 24.1 (31.2) | 10.1 (26.4) | 9.0 (22.0) | 0.034* | 0.500 | 0.031* | 0.440 |
| *Lactobacillus jensenii* | 5.0 (9.3) | 0.1 (0.3) | 0.0 (0.0) | 0.007** | 0.004*** | 0.007** | 0.315 |
| Uncl. *Lactobacillus* | 0.5 (0.4) | 0.2 (0.3) | 0.2 (0.4) | 0.100 | 0.500 | 0.054 | 0.409 |
| *Limosilactobacillus vaginalis* | 0.1 (0.3) | 0.1 (0.3) | <0.1 (0.1) | 0.833 | 0.353 | 0.500 | 0.280 |
| Other *Lactobacillus* | 0.1 (0.3) | <0.1 (0.1) | 0.0 (0.0) | 0.260 | 0.500 | 0.086 | 0.053 |
| Other | 10.4 (8.5) | 24.8 (33.1) | 47.1 (20.0) | 0.015* | 0.238 | 0.002** | 0.011* |

^a^ Overall: Kruskal-Wallis test; pairwise: Dunn’s post-hoc test

* p<0.05; ** p<0.01; *** p<0.005
